# Successful management of neonatal alloimmune thrombocytopenia in the second pregnancy: a case report

**DOI:** 10.1590/S1679-45082014RC2729

**Published:** 2014

**Authors:** Fabiana Mendes Conti, Sergio Hibner, Thiago Henrique Costa, Marcia Regina Dezan, Maria Giselda Aravechia, Ricardo Antonio D'Almeida Pereira, Andrea Tiemi Kondo, Élbio Antônio D'Amico, Mariza Mota, José Mauro Kutner

**Affiliations:** 1 Hospital Israelita Albert Einstein, São Paulo, SP, Brazil; 2 Universidade de São Paulo, São Paulo, SP, Brazil

**Keywords:** Thrombocytopenia, Blood platelets, Thrombocytopenia, neonatal alloimmune, Infant, newborn, Case reports

## Abstract

Neonatal alloimmune thrombocytopenia is a serious disease, in which the mother produces antibodies against fetal platelet antigens inherited from the father; it is still an underdiagnosed disease. This disease is considered the platelet counterpart of the RhD hemolytic disease of the fetus and newborn, yet in neonatal alloimmune thrombocytopenia the first child is affected with fetal and/or neonatal thrombocytopenia. There is a significant risk of intracranial hemorrhage and severe neurological impairment, with a tendency for earlier and more severe thrombocytopenia in subsequent pregnancies. This article reports a case of neonatal alloimmune thrombocytopenia in the second pregnancy affected and discusses diagnosis, management and the clinical importance of this disease.

## RESUMO

A púrpura trombocitopênica neonatal aloimune é uma doença grave, na qual a mãe produz anticorpos contra antígenos plaquetários fetais herdados do pai, e é ainda subdiagnosticada na prática clínica. É considerada o equivalente plaquetário da doença hemolítica do recém-nascido, com a diferença que o primeiro filho é afetado, apresentando trombocitopenia fetal e/ou neonatal. Há risco significativo de hemorragia intracraniana e sequelas neurológicas graves, com tendência a trombocitopenia mais grave e mais precoce nas gestações subsequentes. Este artigo relata um caso de trombocitopenia aloimune neonatal na segunda gestação afetada e discute diagnóstico, manejo e importância clínica dessa doença na prática clínica.

## INTRODUCTION

Neonatal alloimmune thrombocytopenia (NAIT) is a disease in which the mother produces antibodies against fetal platelet antigens inherited from the father and which the mother lacks.^(1–3)^ It is the platelet counterpart of the RhD hemolytic disease of the fetus and newborn. However, NAIT affects the first pregnancy and can cause intracranial hemorrhage (ICH), with a tendency for earlier and more severe thrombocytopenia in subsequent pregnancies.^(1–5)^

We report the case of a 37-year-old woman with NAIT diagnosed in her first child and the strategy used to manage this second at-risk pregnancy.

## CASE REPORT

A 37-year-old Caucasian female from São Paulo, Brazil, gave birth to a healthy male baby on February 2009 by vaginal delivery (40 weeks) weighing 3510g, Apgar 9 to 10 and no obstetric complications. In less than 24 hours of life, the newborn presented with petechiae and severe thrombocytopenia (14,000/mm^3^), despite normal hemoglobin and white blood cell (WBC) counts (Table 1) and absence of infection. The baby was transferred to the neonatal intensive care unit (NICU) for investigation.

**Table 1:** Hematimetric parameters of the first newborn until discharge

	2/26 D1	2/27 D2	2/28 D3	3/2 D5	3/3 D6	3/4 D7	3/5 D8	3/6 D9	3/8 D11
Erythrocytes (mm^3^)	5.05	4.89	4.16	3.57	3.79	3.32	3.31	3.28	3.27
Hemoglobin (g/dL)	16.5	15.6	13.9	11.3	12.4	10.6	11.1	10.5	10.3
Hematocrit (%)	49.5	48.2	41.9	35.8	38.1	32.6	33.5	31.2	31.6
MCV (fL)	97.6	98.6	100.7	100.3	100.5	98.2	99.7	95.1	96.6
MCHC (g/dL)	33.4	32.3	33.2	31.7	32.5	32.5	33.6	33.8	32.7
RDW (%)	15.1	16.5	15.7	15.0	15.8	15.5	14.9	15.6	16.6
Leucocytes (mm^3^)	37,200	25,500	19,700	12,900	18,300	19,000	17,800	14,000	12,000
Platelets (mm^3^)	14,000	21,000	20,000	9,000	16,000	-	27,000	51,000	81,000

MCV: mean corpuscular volume; MCHC: mean corpuscular hemoglobin concentration; RDW: red cell distribution width.

The platelet count reached its lowest level on day 4 (9,000/mm^3^), despite daily platelet transfusions and IV immunoglobulin 1g/kg. On day 8, platelets finally raised to 51,000/mm^3^ and the baby was discharged with 81,000/mm^3^ on day 9, without any bleeding complications.

**Figure 1:**
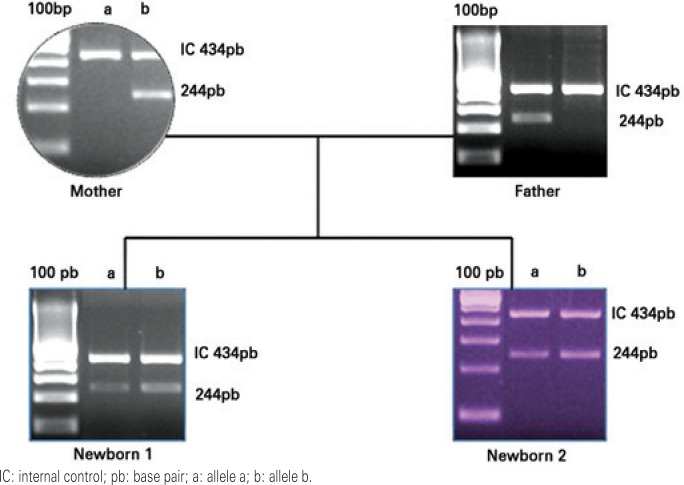
Summary of genotyping results from mother, father and first and second child

Human platelet antigen (HPA) genotyping showed that the mother was HPA-1b1b, the father HPA-1a1a and the child HPA-1a1b (Figure 1). Maternal antibodies against HPA-1a were detected by monoclonal-specific antibody immobilization of platelet antigens (MAIPA), confirming the diagnosis of NAIT.

In March 2012 this patient became pregnant again. The sibling was stratified to a standard risk of bleeding and intravenous immunoglobulin (IVIG) 1g/kg/week was started at week 17. Regular ultrasound scans were performed to monitor fetal ICH. At week 20, MAIPA was performed on the mother's serum and confirmed the anti-HPA-1a.

**Figure 2:**
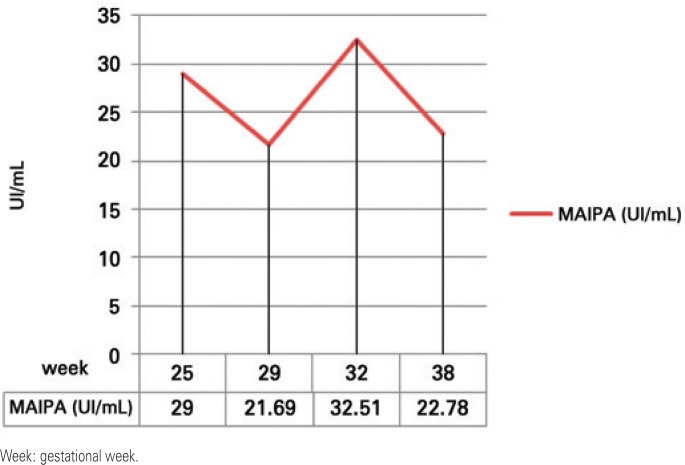
Anti-HPA-1a titer curve in mother serum during second pregnancy by quantitative monoclonal-specific antibody immobilization of platelet antigens (MAIPA)

Non-invasive follow-up with quantitative MAIPA was used to assess the risk of neonatal thrombocytopenia instead of cordocentesis. It was performed at weeks 25, 29 and 32, and the results were 29UI/mL, 21.69UI/mL and 32.51UI/mL, respectively (Figure 2). Oral prednisone 40mg/day was started at week 32 and C-section was chosen to minimize the risk of bleeding at delivery. Moreover, HPA-1b1b donors were scheduled for plateletpheresis donation close to the estimated date of delivery.

The mother had moderate anemia during pregnancy (lowest level Hb=8.7g/dL at week 36). Hemolytic anemia due to IVIG was excluded by normal lactate dehydrogenase (LDH) levels (383mg/dL) and negative direct antiglobulin test. Since iron (87μg/dL) and ferritin (47.9ηg/mL) levels were normal, anemia was considered dilutional and IVIG was not interrupted.

Although delivery was scheduled for week 38, the woman went into labour and the baby was born at week 37, weighing 2750g and with Apgar 9 to 10, without petechiae or ecchymoses and a platelet count of 59,000/mm^3^. He remained in the neonatal ICU for close monitoring. Intracranial and abdominal ultrasound scans were normal. On day 2, he had 99,000/mm^3^ platelets and was discharged on day 3, asymptomatic with platelet count of 150,000/mm^3^. Platelet transfusions were not necessary.

## DISCUSSION

NAIT affects 1:1000 live births, and in severe cases, evolves to ICH with severe neurological impairment in 10 to 22% of children, and 75% of bleeding occur antenatally.^(4)^ Despite being the most frequent cause of severe thrombocytopenia in fetuses and neonates, and the most frequent cause of ICH in the newborn,^(4)^ it is underdiagnosed in routine clinical practice, with only 37% of severe cases detected in the absence of antenatal screening.^(2)^ Antibodies against HPA-1a account for more than 80% of cases in Caucasians; anti-HPA-3a, −4a and −5a have also been reported.^(1)^ Mothers have the rare HPA-1b1b genotype, whereas the fetus inherit a HPA-1a allele from the father.

NAIT is suspected in a fetus with intracranial bleeding on ultrasound and neonates with bleeding or severe unexplained and/or isolated thrombocytopenia. The mother has normal platelet counts and no history of autoimmune disease or drug-induced thrombocytopenia.^(1–3)^

Testing for fetal and neonatal alloimmune thrombocytopenia (FNAIT) should be performed in any neonate with unexplained thrombocytopenia <50.000/mm^3^, regardless of the presumed cause.^(2)^ Diagnosis is made by platelet genotyping showing HPA fetal-maternal incompatibility and detection of anti-HPA antibodies in the mother's serum.^(1)^

There is a tendency for non-invasive monitoring during pregnancy, considering that 1.3% of fetal death cases per procedure and 5.5% cases per affected pregnancy were attributed to cordocentesis.^(2,5–8)^ The predictive value of the quantitative MAIPA for neonatal thrombocytopenia was reported by Bertrand et al.^(9)^, in 2005, and results were confirmed by Killie et al., in 2008.^(10)^ The exact role of sequential quantitative anti-HPA measurements remains to be established, but antibody clearance along pregnancy seems to have a better prognosis.^(11)^

The maternal treatment of NAIT^(2,4–8,11–14)^ is based on the outcome of the first affected child and is classified into: (a) standard risk: first child with platelet >20.000/mm^3^ and no bleeding history; (b) high risk: platelet <20.000/mm^3^ or neonatal bleeding; (c) very high risk: fetal bleeding between 28 and 36 weeks; and (d) extremely high risk: fetal bleeding before 28 weeks. Weekly IVIG infusions are the treatment mainstay, varying in dose (1 to 2g/kg/week) and time to start (20 weeks or earlier). High-risk mothers receive higher IVIG doses and oral prednisone 0.5-1mg/kg, starting at 16 weeks for very high risk or 12 weeks for extremely high risk pregnancies.^(4,6,12)^ C-section seems to be safer than vaginal delivery, especially when the fetal platelet count is unknown.^(2,5,6,14)^

HPA-matched platelet transfusions are indicated during the first 24 hours of life if platelets <30.000/mm^3^ or if there are signs of bleeding.^(5,15)^ IVIG alone is not recommended, as it takes 24 to 48 hours to be effective with risk of central nervous system (CNS) bleeding^(5)^ HPA-1b1b plateletpheresis donors should be contacted to donate close to the estimated date of delivery. Alternatively, the mother can donate platelets if feasible.^(15)^ IVIG 1g/kg for 2 days can be associated to platelet transfusions to the newborn in severe cases.^(5,15)^

## CONCLUSION

Neonatal alloimmune thrombocytopenia is a serious underdiagnosed condition that affects the first child associated with significant morbidity. Close monitoring during pregnancy with ultrasound scans, treatment with immunoglobulin IV and/or corticosteroids and HPA-matched platelets at delivery can provide proper medical support to this condition and reduce the incidence of neurological sequelae in the newborn.
